# Re-using food resources from failed honey bee (*Apis mellifera* L.) colonies and their impact on colony queen rearing capacity

**DOI:** 10.1038/s41598-023-44037-2

**Published:** 2023-10-23

**Authors:** Rogan Tokach, Autumn Smart, Judy Wu-Smart

**Affiliations:** Department of Entomology, University of Nebraska-Lincoln, Lincoln, NE 68583 USA

**Keywords:** Environmental impact, Ecology, Environmental sciences, Environmental impact

## Abstract

For over a decade, beekeepers have experienced high losses of honey bee (*Apis mellifera* L.) colonies due to a variety of stressors including pesticide exposure. Some of these chemical stressors may residually remain in the colony comb and food resources (pollen and nectar) of failed colonies and be later re-used by beekeepers when splitting and building back new colonies. The practice of re-using comb from previously perished colonies (termed “deadout”) is common in beekeeping practice, but its role in affecting colony health is not well understood. Here, we evaluate the impact of reused, pesticide-contaminated “deadout” combs on colony function during the process of replacing a queen bee. Queenless microcolonies were established to monitor queen rearing capacity in two treatment groups: (1) colonies given frames containing food resources from deadout colonies in control “clean” apiaries and, (2) colonies given frames containing “contaminated” resources from deadout colonies originating from apiaries experiencing chronic pesticide exposure from widespread systemic pesticide pollution (including neonicotinoid insecticides: clothianidin and thiamethoxam). Results indicate that colonies given pesticide-contaminated resources produced fewer queen cells per colony and had a lower proportion of colonies successfully raising a functional, diploid egg-laying queen. This research highlights the deleterious effects of re-using deadout combs from colonies previously lost due to pesticide contamination.

## Introduction

Honey bees (*Apis mellifera* L.) are pollinators of over 58 different agricultural crops^1^, and these pollination services are valued at $34 billion USD annually in the United States^2^. Their pollination services are also vital for trees, shrubs and wildflowers that contribute to biodiversity across various ecosystems^3^. The health and status of managed pollinators continues to garner much attention. Rather than a singular stressor, numerous factors have been associated with declining honey bee colony health, including poor nutrition, parasites and diseases, and exposure to pesticides^4–6^. Many of these stressors are known to interact in conjunction with each other to ultimately induce colony failure^6–9^.

Honey bee colonies rely on the availability of resources in their surrounding environments to support their populations, produce honey crops, and survive through both short and long-term dearth. Due to their foraging behaviors and ecological services, bees are often utilized as a biological indicator species in remote sensing and ecological modeling^10–12^. For example, monitoring the populations, weight changes, and temperatures of colonies can provide researchers with environmental quality information and alert them of potential pesticide exposure issues^12,13^.

Pesticides are necessary in agriculture for use in control of insect pests, weeds, and against crop diseases^14^, and recent trends suggest a reduction in insecticide applications on cropland in the US^15^. However, since 2015, nationally derived and reported pesticide use data no longer includes data for seed treatments including systemic, water-soluble insecticides and fungicides designed to translocate throughout a plant resulting in protection against pests, especially during early stages of growth^16^. A majority of conventional crops grown in the Midwestern US (e.g. corn, soy, canola) utilize pesticide-treated seeds at planting^17^. However, only 1.6–20% of the active ingredient(s) on treated seed is taken up and absorbed by the target crop, while the remaining residues may persist in the soil, leach into groundwater, or may be translocated into non-target, nearby plants^18–20^. Bees may become exposed to systemic pesticide residues during foraging through contaminated nectar, pollen, and water sources^21–23^. Pesticide-laden resources may be directly consumed by bees or stored in comb cells thus potentially exposing nestmates, including workers, immature brood, and reproductive individuals (queen and drones) within the colonies^24–26^.

Beekeepers that observe depopulation of worker bees or complete colony failure commonly reintegrate and combine previously used resources (i.e., used comb, brood, and food resources) to boost weak colonies or start new colonies by restocking the worker population and providing a laying queen^27,28^. Pesticide residues in food stores and comb cells may accumulate over time, increasing the number and levels of active ingredients and metabolites and increasing the risk of adverse interaction effects from chemical mixtures and with other non-chemical stressors^25,26,29,30^. For example, honey bee workers reared in pesticide-contaminated wax comb exhibit shorter lifespans and greater susceptibility to parasites and pathogens^31,32^. Therefore, while the practice of reusing comb may benefit colonies by adding resources and reducing the load of pesticide residues and other disease agents, the practice may also have detrimental impacts to colony health, particularly if the comb originated from failed colonies.

Numerous studies have demonstrated the lethal and sublethal effects of pesticides, particularly insecticides, on all castes of the honey bee^33–38^. Acute pesticide exposure can result in individual bee death or losses of entire colonies^36,39^. Exposure to modern agricultural pesticides, such as systemic neonicotinoid insecticides, more commonly results in sublethal effects on colony functioning. Oral and contact exposure impacts cognitive and locomotor processes such as memory retention, learning capacity, and flight navigation that are critical for foraging and nestmate interaction^38,40–42^. Other colony-level sublethal effects of pesticides include induction of precocious foraging, reduction of hygienic behavior, and physiological and morphological changes when exposure occurs during development^43,44^.

Honey bee queens exposed to neonicotinoids have been shown to exhibit reduced egg laying, physiological differences in enzyme activity in response to stressors, and reduced mating efficiency when exposed both before and after adulthood^38,45–47^. Drones exposed to pesticides during development and as adults have been shown to have reduced sperm viability and increased fertility impairment^35,48^. Pesticide impacts on queens and drones have colony-level ramifications; colonies with nonproductive or failing queens must quickly respond by engaging in the queen-rearing process or risk becoming a queenless colony which eventually depopulates, weakens, and dies. Multiple factors may contribute to queen failure, identified as a colony lacking a mated egg-laying queen, production of emergency or supersedure cells, over-production of male offspring, or the presence of a new virgin or replacement queen. Factors that correlate with queen failure include poor mating success, mismanagement of colonies, pesticide exposure, parasites, pathogens, and combinations of such factors^49^. While a primary cause of queen failure has yet to be identified, correlation between the frequency of queen failure events and pesticide residue contamination in beeswax and pollen stores has been shown^30,50^.

When queenless, colonies feed young, diploid larvae a specialized diet of protein-rich secretions synthesized in nurse bees’ hypopharyngeal and mandibular glands (i.e., royal jelly) to trigger development of reproductive queens^51–53^. Subsequent to queen loss, production of a new queen must occur within a short developmental period (when approximately 0–3-day old larvae are still present) to ensure a high quality queen emerges 16 days later from its “queen cell”, which houses the queen during immature development^54^. Feeding royal jelly to very young larvae (< 1 day old) results in higher quality queens as measured by morphological features such as heavier weight and larger thorax width^55^. Queens exhibiting these characteristics also have an increased mating number, stored sperm count, and percentage of their spermathecae filled^56^. Higher quality queens subsequently produce stronger colonies with greater wax production, food stores, and improved colony survival^55^.

Pesticides, in addition to playing a role in outright queen failure, can also impact the success of queen replacement (requeening) and quality of queens produced. Because both adult and immature queens are directly fed by nurse bees, nurses act as a colony buffer by essentially filtering out pesticides from the food fed to queens. However, the protein composition of royal jelly originating from nurse bees feeding on pesticide-contaminated pollen may be altered by a reduction in the levels of several key nutrients that may, in turn, lead to a reduction in queen quality^57^. Further, colonies fed pollen containing field-relevant pesticide levels have been shown to have lower adult queen emergence and produce fewer queen cells, while the queens that emerge and mate may have less viable spermatozoa stored in their spermathecae^58,59^.

Currently, pesticide-treated seeds are classified as “treated articles,” and due to federal exemptions, regulatory oversight only occurs at the seed factory when the chemicals are initially applied to the crop seeds. Once the treated seeds enter the market, they are no longer subject to the rigorous rules and guidelines that all other pesticide applications (foliar, chemigation, injection) are required to follow under the Federal Insecticide, Fungicide, and Rodenticide Act^60^. The discrepancy in regulation over pesticide-treated seeds has led to concerns over their widespread use in agricultural and urban landscapes as well as proper disposal of pesticide-treated seeds when excess, expired seed becomes unviable, but the chemicals nevertheless remain active. Large scale seed disposal recommendations include disposal in landfills, high-temperature incineration, use as a fuel source for power plants, or in fermentation processing at an ethanol plant^61^. Beginning in 2015, AltEn LLC, an ethanol plant located in Mead, Nebraska began stockpiling and using expired, pesticide-treated seed as a primary source of carbohydrates for ethanol production. As a result, liquid effluent and solid waste byproducts heavily contaminated with pesticide residues were produced in large volumes and sold as soil conditioners or amendments to nearby farmers^62^. In 2019, the Nebraska Department of Agriculture (NDA) prohibited distribution of the distiller grain byproduct after pesticide tests found that land application at the recommended amount would have resulted in a rate 85 times higher than the allowed in a typical pesticide label application of clothianidin^62^. The plant subsequently continued to produce and stockpile waste byproduct on site, which contained high levels of numerous pesticides, leading to further wide-scale pollution near the facility where pesticide particles were released into the air, soil, and water^62^. University of Nebraska-Lincoln apiaries located within 0.5–2.0 km (~ 1 to 3 mi.) of the ethanol plant suffered 100% colony failure from 2019 until the plant was shut down in 2021 (Table 1).

**Table 1:** University of Nebraska–Lincoln Bee Lab colony loss summary (% mortality).

Site	Year		
	2019	2020	2021
ENREEC	100 (n = 20)	100 (n = 12)	69.4 (n = 23)
Kimmel Orchard	0 (n = 4)	25 (n = 4)	50 (n = 4)
Pollinator Garden	0 (n = 4)	0 (n = 4)	25 (n = 4)

Colony mortality percentage from the ENREEC pesticide laden environment compared to two control sites over 3 years (n = number of colonies).

This study asks whether the common management practice of reusing comb and food stores from colonies that previously failed or died due to suspected pesticide exposure in a contaminated landscape may impact the ability of new colonies to rear queen honey bees compared to colonies set up with resources from colonies managed in a more typical, unpolluted landscape in the Midwestern U.S. region. This study had two hypotheses:

Hypothesis 1. Number of Queen Cells Produced Per Colony: Colonies provided frames of real-world, pesticide-contaminated resources (“contaminated” comb treatment) will produce fewer queen cells per colony compared to those given relatively uncontaminated resources (“control” comb treatment).

Hypothesis 2. Number of Colonies Successfully Requeened: Fewer contaminated treatment colonies will successfully requeen themselves with new, functional (diploid egg-laying) queen relative to those in the control treatment.

## Results

### Queen cell production

The total brood area given to each queenless nucleus colony did not differ based on treatment (F_1,8_ = 3.14; p = 0.11), site (F_2,5_ = 0.61; p = 0.58) or treatment by site interaction (F_2,8_ = 1.55; p = 0.27), signaling colonies received approximately the same amount of eggs and young larvae from the outset of each replication. Additionally, the site locations where the experiment was carried out (UNL Pollinator Garden, ENREEC, Kimmel Orchard) did not impact the number of queen cells produced per colony (F_2,5_ = 4.69; p = 0.07), nor did the interaction between site and treatment (F_2,8_ = 0.50; p = 0.62).

**Figure 1:**
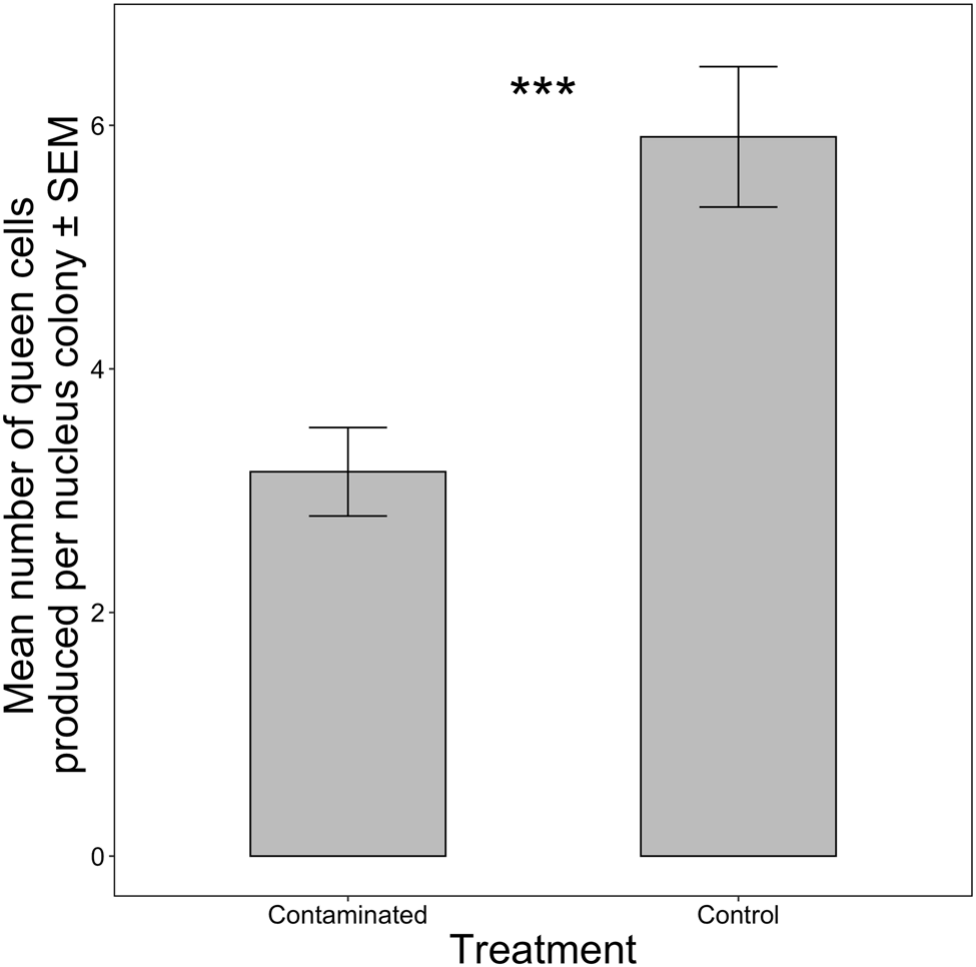
Queen cell production. Mean number of queen cells produced per nucleus colony ± standard error of mean (SEM) based on treatment of resources (“control” vs “contaminated”) given to the colonies. Significant differences denoted by *p ≤ 0.1, **p ≤ 0.05, ***p ≤ 0.01 (comparison with Controls).

Colonies in the control group produced significantly more queen cells compared to those colonies given contaminated resources (Fig. 1: mean_control_ = 5.9, mean_contaminated_ = 3.2, F_1,8_ = 17.24; p < 0.01).

### Requeening rate

**Figure 2:**
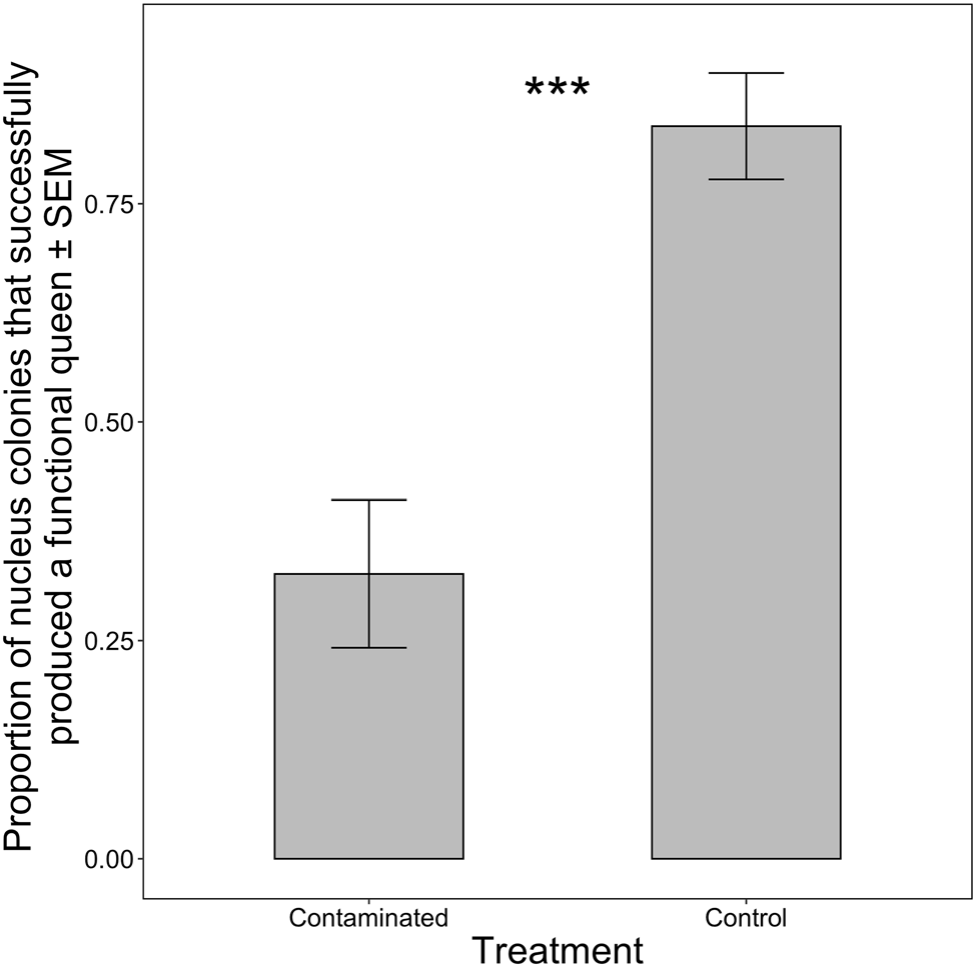
Nucleus colony requeening success. Proportion of nucleus colonies ± standard error of mean (SEM) to successfully produce a functional laying queen based on treatment of resources given to the colonies. Significant differences denoted by *p ≤ 0.1, **p ≤ 0.05, ***p ≤ 0.01 (comparison with Controls).

Of the queen cells that were produced, not all successfully reached adulthood or became viable laying queens. Contaminated treatment colonies resulted in significantly fewer viable requeening events, producing a mated, worker brood-laying queen successfully 32.6% of the time compared to control colonies with an 83.9% viable queen production rate (Fig. 2: F_1,8_ = 19.05; p < 0.01). There was no statistical difference in the proportion of viable queens produced by site (F_2,5_ = 0.31; p = 0.75) or for the treatment by site interaction (F_2,8_ = 0.12; p = 0.89). We also examined whether the number of queen cells produced by a colony was related to requeening success. The contaminated treatment colonies that successfully requeened themselves produced on average 4.2 queen cells per colony while contaminated treatment colonies that failed to requeen themselves produced on average 3.0 queen cells per colony, however, the difference was not statistically significant (F_1,80_ = 2.09; p = 0.15).

## Discussion

Our study shows colony resources from pesticide-contaminated hives can hinder workers’ ability to successfully rear queens, meaning natural requeening may not be feasible in such instances. Colonies given contaminated food stores produced fewer queen cells compared to their control counterparts, and a lower proportion of those colonies were able to successfully requeen themselves. There was not a difference in queen cell production or requeening success seen between sites (UNL pollinator garden, ENREEC, Kimmel Orchard), suggesting the provisioned experimental frames (and resources contained therein) were the primary factor driving queen rearing success. Specifically, contaminated pollen stores were likely the driving force leading to the reduction in queen rearing capacity since honey from the pesticide contaminated colonies only contained trace levels of residues. This discrepancy in pesticide loads detected in stored pollen versus honey is likely due to the differences in their collection method because foragers must consume the contaminated nectar and likely do not return to the hive but rather die or become impaired during collection as opposed to pollen which is not consumed but rather packed onto their legs and carried back to the hive without consumption.

Although many commercial beekeepers have mated queens readily available, colonies that prematurely lose their queen or swarm still rely on natural requeening methods. Some beekeepers also practice “walk away splits” which divides the brood and resources and relies on the queenless portion of the split to naturally requeen as a form a swarm management. Throughout all levels of beekeeping, queen failure continues to be self-reported as a prominent reason for colony failure. Queen failure is a common beekeeper-reported cause of colony loss, most notably for hobbyist beekeepers but also for those at the commercial and sideline levels^63^. Further, increased incidence of queen events correlates with known colony pesticide exposure^30^.

While the number of queen cells produced naturally varies^51,54,64^, the reduced number of queen cells observed in the contaminated treatment colonies corresponds with previous work showing that nucleus colonies fed pesticide-contaminated pollen had a lower number of queen cells constructed^58^. For the queen rearing process, queen breeders use strong colonies containing large workforces and food stores as cell builders because of their ability to produce and support a higher number of queen cells^65^. Colonies compromised by stressors such as pesticide exposure are relatively weakened, limiting their queen cell production potential. Quality of developing queen larvae is also a driving factor in the number of queen cells produced per colony. Worker bees may remove or abort queen cells with developing larvae or pupae that they deem unfit^66^. Though direct observations of worker bee interactions with queen cells were not evaluated in this experiment, the number of queen cells could have been reduced due to an increased rate of queen cell removal by worker bees.

Coinciding with previous work^58,59,67,68^, this study demonstrated that queen-rearing capacity and production of functional queens were impacted by pesticides, but further, we show that the practice of reusing colony resources from deadout colonies can have detrimental colony-level ramifications (Fig. 2). In our experiment, nurse bees and brood from the same mother colonies were used, ensuring any prior pesticide exposure or other stress factors were equalized among colonies before placement into experimental conditions. Preceding studies have shown reduction in queen viability when colonies were exposed to pesticide treatments for 28 days or more to ensure an entire brood cycle had been reared in contaminated conditions^58,59,67^. We observed comparable results with workers not originally fed pesticide contaminated resources during larval development. This result signifies that a shorter, more limited pesticide exposure in our contaminated treatment colonies was still enough to significantly impact colony queen-rearing abilities.

The relatively rapid negative impact on queen rearing seen in this study may have been influenced by the size of colony used. Honey bee colonies consisting of a large workforce can act as buffers to pesticide toxicity via trophallaxis to disperse and dilute pesticides throughout their population, potentially limiting potency and sublethal impact on colonies^38^. Our study used nucleus colonies containing relatively small populations, thus potentially increasing the relative pesticide dosage to each individual bee. Queen breeders often establish small nucleus colonies in which to place queen cells for adult emergence and subsequent mating^65^. While workers in these mating nucleus colonies do not feed developing queen larvae, the reduced workforce is still responsible for feeding and attending the adult queen post-emergence, leaving them relatively more susceptible to pesticide exposure due to the limited buffering capacity of small colonies. Typical spring colonies in the midwestern U.S. (overwintered colonies, package bees, or splits) are also comprised of a relatively small number of workers and, further, coincide with treated seed crop planting. Research investigating colony size, pesticide exposure under field conditions, and timing is needed to determine when buffering capacity may or may not affect queen rearing success.

There are multiple potential factors affecting reduced viability of queens when reared in pesticide-contaminated environments. Although nurse bees can act as a buffer limiting the transmission of toxicants from pollen into royal jelly^57,68–70^, the nutritional composition of royal jelly protein may be compromised when workers are exposed to contaminated pollen^57^. Additionally, pesticide exposure during larval development or from consumption as an adult can cause deterioration of hypopharyngeal glands in workers and negatively impact their royal jelly production^71–74^. Further, queens reared in pesticide laden wax may have reduced egg-laying rates and altered components of queen mandibular gland secretions, which can lead to impacts on retinue attendance^75^. While this study was short-term and did not address royal jelly production, composition, hypopharyngeal gland size or worker behavior, there is an interesting opportunity for future work to investigate their relation to queen rearing success.

Healthy and productive queens are imperative for a successful colony to withstand stress^55^. Based on this study’s results, beekeepers reusing deadout combs and resources may be at risk of harming their colonies by exposing them to additional pesticides, increasing their risk of queen events^30^, and limiting their ability to produce functional queens. Beekeepers should refrain from reusing deadout resources if pesticide-associated colony failure is suspected or until a necropsy has been conducted on the colony^76^. Resources from colonies deemed pesticide kills should be disposed of and not recycled into active colonies^77^. Additionally, comb rotation should be considered for beekeepers to remove accumulated pesticides found in wax and resources, especially those intended for use in queen rearing^78,79^. Our findings elucidate an environmental component impacting queen rearing ability in honey bee nucleus colonies. This study highlights the importance of tracking frames from deadouts and the potential harm that can occur when colonies are built back using old comb and resources. Colonies located adjacent to agricultural settings may have an increased risk of pesticide exposure which can prove limiting to queen rearing success. Our work supports the body of literature demonstrating that pesticide exposure can weaken queen rearing and contribute to colony failure. Continued work examining the causes of queen rearing failure can aid in shaping beekeeping best management practices in the future.

## Methods

### Colony setup and comb treatments

**Figure 3:**
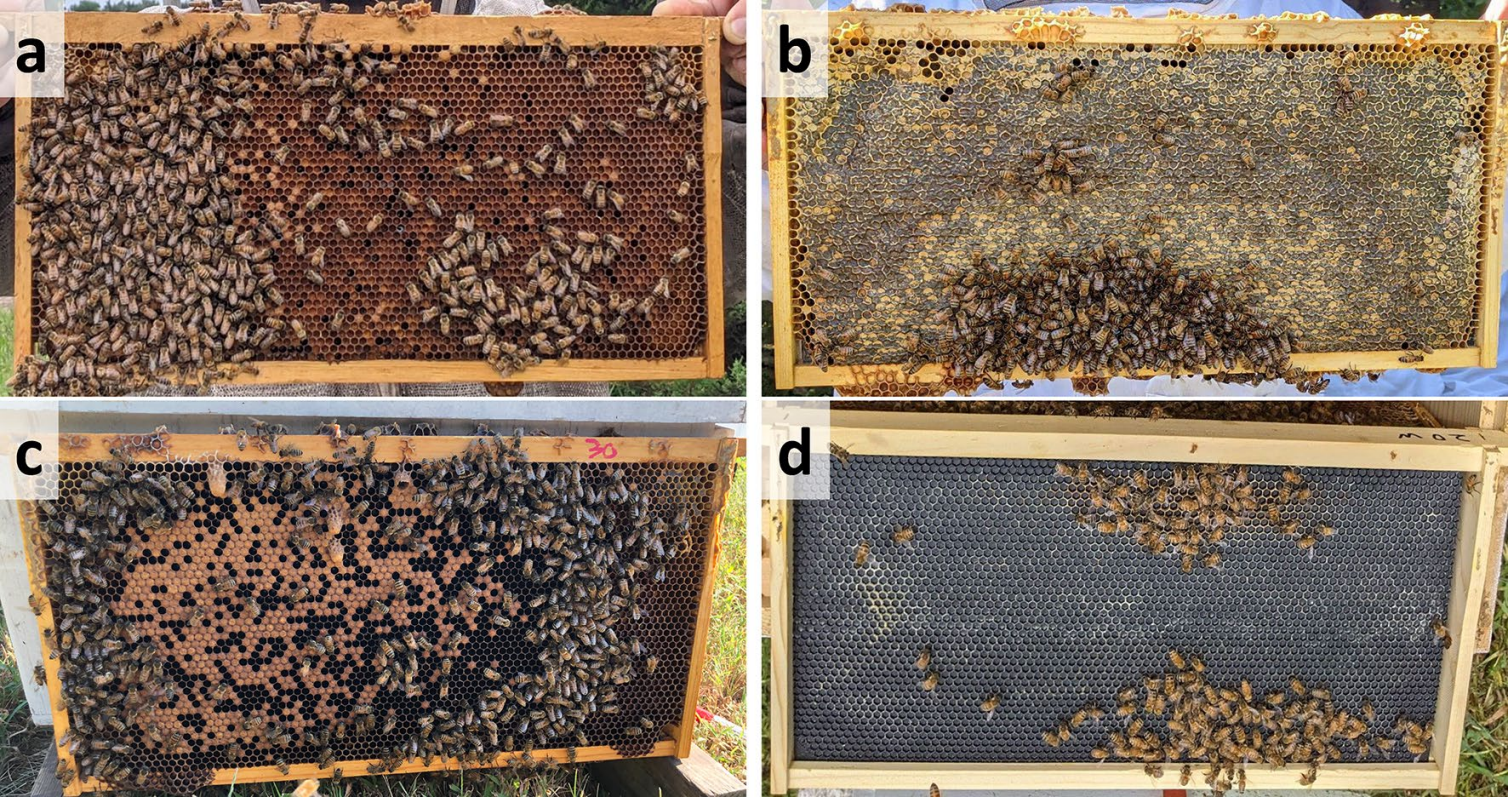
Experimental nucleus colony set-up. Examples of the four experimental frames given to individual nucleus colonies. Resource frames containing pollen stores (**a**) and honey or nectar stores (**b**), a frame with eggs (**c**), and an empty foundation frame (**d**). The egg frame shown is from 1 week after colony establishment and developing worker and queen cells are visible.

Each experimental nucleus colony was supplied with four standard Langstroth frames containing comb cells; two frames contained capped honey and pollen stores from deadouts either originating from “contaminated” (colonies located near the ethanol plant) or control (colonies with minimal pesticide exposure) apiaries, one frame containing 1-day-old eggs extracted from healthy colonies managed in the control apiary, and one frame of foundation (Fig. 3). Each colony was supplied with approximately 3500 adult worker bees from a common pool of colonies and no queen. All egg frames and adult worker bees originated from existing UNL Bee Lab colonies located in the pollinator garden apiary (Lincoln, NE). These colonies were routinely monitored and treated for *Varroa* mites to prohibit mite infestation from impacting queen production. Egg frames had, on average, 31.9% of each side containing eggs or young larvae, or approximately 1000 individuals from which to rear replacement queens. Resource frames (capped honey and pollen or bee bread (Fig. 3a,b) for control colonies were taken from colonies that had failed during the previous 2019, 2020, or 2021 winter but did not exhibit pesticide or disease stress symptoms. During the winter, prior to use, these frames were stored indoors with wax moth crystals to prevent damage. Treatment (contaminated) colonies were given food resource frames from 2018 or 2019 deadout colonies that exhibited classic acute pesticide toxicity and elevated worker mortality over several months after being placed in apiaries near the ethanol facility. These frames were stored in a freezer at − 20 °C until use in these experiments to prevent pesticide residue degradation. Once removed from the freezer, these frames were placed in lidded Langstroth deep boxes to prevent light degradation^80^ and to thaw for 1 day before being placed in nucleus colonies. Pesticide analyses of pollen stores collected from control combs measured only trace amounts of 12 different pesticides. To contrast, the number of detected pesticides in contaminated treatment pollen (tested in 2019 and 2020) ranged from 5 to 16 compounds and two compounds in particular, clothianidin and thiamethoxam, were present in all contaminated pollen. While there is a debate on what Hazard Quotient (HQ) level should be determined significant risk^81^, HQ levels for these two compounds exceeded all risk threshold levels (Table 2). Honey stores from contaminated combs were also tested, but only trace amounts of pesticides were detected.

**Table 2:** ENREEC pollen pesticide levels.

Insecticide^a^	Detects	% of samples	Mean (ppb)	Max HQ	Mean HQ	Samples above 1000 HQ
Clothianidin	11	100	109.3	71,125.0	27,313.6	11
Thiamethoxam	11	100	25.6	10,180.0	5125.5	11
Thymol^b^	2	18.2	484.5	3.6^c^	2.4	0
Chlorantraniliprole	10	90.9	20.6	0.4	0.2	0

Pesticide residue levels and hazard quotients found in pollen samples taken from colonies on ENREEC property (n = 11 pooled samples).

^a^11 fungicides and 2 herbicides also detected with minimal HQ effect.

^b^Thymol was not tested for in 9 of 11 samples.

^c^Thymol contact LD_50_ was used for HQ calculation instead of oral LD_50_.

### Apiary sites

We used a total of 104 small nucleus colonies distributed among 4 replicates over the course of 3 years from 2020 to 2022 for our experiments. Nucleus colonies were set up and equally distributed for replication 1 among two sites in 2020, (1) the University of Nebraska-Lincoln (UNL) pollinator garden located on the UNL East Campus in Lincoln, Nebraska, and (2) Kimmel Orchard located in Nebraska City, Nebraska. Replications 2–3 were conducted in 2021 and replication 4 occurred in 2022. These replications (2–4) utilized three sites, including (1) the UNL pollinator garden, (2) Kimmel Orchard, and (3) the East Nebraska Research Extension and Education Center (ENREEC) located near Mead, Nebraska. These sites were an average of 91 km apart in distance. Replication 1 used eight colonies of each treatment per site resulting in a total of 32 colonies, 16 per site. Subsequent replications used four colonies of each treatment per site totaling 24 colonies, eight per site (Table 3).

**Table 3:** Experimental colony arrangement from 2020 to 2022.

Year	Replication number	Number of colonies		
		ENREEC	Kimmel	Pollinator Garden
2020	Rep 1	N/A	16	16
2021	Rep 2	8	8	8
2021	Rep 3	8	8	8
2022	Rep 4	8	8	8

Number of colonies at each site based on replication with colonies evenly split between two treatments.

### Colony inspections and measurements

One week after establishment, colonies were inspected, and photos of egg-containing frames were taken to facilitate counting the total number of queen cells produced per colony (Hypothesis 1). The total brood-containing area was also quantified at this time to discern differences in total brood area between treatments. Subsequently, colonies were checked once a week for the next 4 weeks to determine when queens had emerged and begun laying eggs. Five weeks after initial installation, colonies were given a final inspection to determine whether they had successfully requeened themselves (Hypothesis 2). Requeening was only considered successful if the queen had laid diploid (fertilized) worker eggs, thus colonies with queens incapable of producing fertilized eggs (i.e. drone-laying queens) were not considered successfully requeened.

### Statistical analyses

SAS^®^ 9.4 software (SAS Institute, 2012) was used to perform statistical tests and R (R Core Team 2022) was used for data visualization. To compare queen cell production per colony among treatment and site (Hypothesis 1), a generalized linear mixed model with a negative binomial distribution was used because count data were being analyzed. Model fixed effects included treatment, site, and the treatment by site interaction while replicate, the replicate by site interaction, and replicate by site by treatment interaction were included as random effects. These three random effects were used to account for replicate variability, including variability between sites within each replicate, and variability between the units where the treatments were assigned (colonies) within sites and each replicate. To determine the difference in brood area among treatments and sites, a generalized linear mixed model with Beta distribution was used because the proportion of total brood area from two sides of each single frame of eggs given to each nucleus colony was assessed. The difference in proportion of colonies to successfully requeen themselves by treatment and site (Hypothesis 2) was calculated using a generalized linear mixed model with a binomial distribution because the binary result of successful requeening event was being assessed. Treatment, site and the site by treatment interaction were considered fixed effects, while replicate, the site by replicate interaction, and the treatment by site by replicate interaction were all included as random effects. Significant statistical differences were denoted at alpha ≤ 0.05.

## Data Availability

The datasets generated and analyzed during the current study are available from the corresponding author on reasonable request.
